# Perception of Neighborhood Safety and Reported Childhood Lifetime Asthma in the United States (U.S.): A Study Based on a National Survey

**DOI:** 10.1371/journal.pone.0006091

**Published:** 2009-06-30

**Authors:** S. V. Subramanian, Malinda H. Kennedy

**Affiliations:** Department of Society, Human Development and Health, Harvard School of Public Health, Cambridge, Massachusetts, United States of America; Universidad Nacional Mayor de San Marcos, Peru

## Abstract

**Background:**

Recent studies have emphasized the role of psychosocial stressors as a determinant of asthma, and neighborhoods can be a potential source of such stressors. We investigated the association between parental perception of neighborhood safety and reported lifetime asthma among children.

**Methodology/Principal Findings:**

Data for the study came from the 2003–04 National Survey of Children Health (NSCH); a nationally representative cross-sectional sample of children aged 0–17 years. Demographic, socioeconomic and behavioral covariates were included in the study. Models were estimated after taking account of weighting and complex survey design. Parental report of whether the child has ever been diagnosed with asthma by a physician was used to define the outcome. Parental report of perception of neighborhood safety was the main exposure. In unadjusted models, the odds ratio (OR) for reporting asthma associated with living in neighborhoods that were perceived to be sometimes or never safe was 1.36 (95% confidence intervals [CI] 1.21, 1.53) compared to living in neighborhoods that were perceived to be always safe. Adjusting for covariates including exposure to second hand tobacco smoke, mother's self-rated health, child's physical activity and television viewing attenuated this association (OR 1.25, 95% CI 1.08, 1.43). In adjusted models, the increased odds ratio for reporting asthma was also higher among those who perceived neighborhoods as being usually safe (OR 1.15 95% CI 1.06, 1.26), as compared to always safe, suggestive of a dose-response relationship, with the differentials for usually safe and never safe being statistically significant (p = 0.009).

**Conclusion:**

Psychosocial stressors may be important risk factors that may impact the pathogenesis of asthma and/or contribute to asthma morbidity by triggering exacerbations through neuroimmunologic mechanisms, as well as social mechanisms.

## Introduction

According to the most recent data an average annual 20 million children and adults in the U.S. had asthma, with a current asthma prevalence of 7.2% [1]. Research on the determinants of asthma has largely investigated the role of environmental exposures, such as exposure to aeroallergens, indoor and outdoor air pollution, endotoxin, smoking, and viral infections [2], [3]. More recently, the role of psychosocial stressors as a determinant of asthma has been proposed [4], [5], [6], [7], [8]. Laboratory as well as prospective population-based studies have shown associations between stress experiences and asthma expression [9], [10], potentially mediated through physiologic pathways resulting in enhanced IgE expression, enhanced allergen-specific lymphocyte proliferation and differential cytokine expression in children [11], [12], [13]. A potential source of psychosocial stress is the type of neighborhood in which people reside, which increasingly has been given important consideration in asthma disparities[14], [15], [16], [17] and epidemiologic research [18], [19], [20], [21], [22]. One hypothesized mechanism through which a neighborhood can induce stress among its residents is through exposure to violence. Residents chronically feel unsafe in these neighborhoods, [23] which leads to increased stress. There are few studies of the association between exposure to neighborhood violence and asthma, [24], [25], [26] with most studies relying on small community samples. We investigated the association between parental perception of neighborhood safety and reported lifetime asthma in a large, nationally representative sample of children aged 5–17 years in the United States (U.S.)

## Methods

### Study population and design

Data for the study came from the National Survey of Children's Health (NSCH) which was a telephone survey conducted in 2003–2004 that gathered information from parents on the physical, emotional and behavioral health of a nationally representative sample of 102,353 children aged 0–17. A full description of the survey can be found at www.cdc.gov/nchs/about/major/slaits/nsch.htm. A complex sample design involving clustering of children within households and stratification of households within states based on the National Immunization Survey sampling plan was used to collect the data. Random digit dialing was used to create the sample in each state. One child per household was randomly selected for enrollment. The survey is representative of non-institutionalized children ages 0–17 at both the national and state levels. Approximately 2000 surveys were collected per state (1,483–2,241) using the SLAITS (State and Local Area Integrated Telephone Survey) program. The SLAITS methodology recognizes the potential for bias resulting from the non-coverage of households without telephones and applies direct adjustments in an attempt to minimize this potential bias [27]. Specifically, in order to adjust for potential bias of under-representation of households without telephones, post-stratification adjustments were made to the NSCH based on evidence which suggests that households with interrupted telephone service are more similar to households without telephones than to households with telephone service. Using data from other national surveys about the proportion of children in households without telephones in each state, weights were increased for completed NSCH interviews in households with interrupted telephone service (see http://www.cdc.gov/nchs/data/series/sr_01/sr01_043.pdf).

The response rate for the survey was 55.3%. This response rate was calculated as the product of the interview completion rate (68.8%), the screener completion rate (“the proportion of known households where a resident reported whether a child lived in the household”) (87.8%), and the resolution rate (“the proportion of telephone numbers that could be positively identified as residential or nonresidential”) (91.6%) [28]. The survey was conducted in English and Spanish. A total of 102,353 children were enrolled in the survey. Of these, 74,264 children between the ages 5 and 17 were eligible for this study. Those with missing information on asthma (*n* = 124), exposure (*n* = 3,834) and covariates (*n* = 18,270) were excluded, yielding a final analytic sample of 54,002 children, constituting 73% of the surveyed population. In the NSCH survey, approximately 9% of respondents were missing poverty data. To account for this, the National Center for Health Statistics provided five publically available datasets containing imputations for the missing poverty values [29]. These data were used for the analysis.

### Outcome

Lifetime asthma was measured using respondent (primary caregiver) reports in the NSCH survey of whether “a doctor or health professional ever told you that the child has asthma”.

### Exposure

Perceived neighborhood safety was measured through one survey question “How often do you feel (Selected Child) is safe in your community or neighborhood?” with the possible responses of never, sometimes, usually, and always. For analysis, the “never” and “sometimes” responses were combined because of low numbers.

### Covariates

Covariates for the study included child's age, gender, a detailed race/ethnicity variable, a measure of household income/poverty (<100% of poverty level, 100-<200% of poverty level, 200-<400% of poverty level, and ≥400% of poverty level), highest level of education obtained by anyone in household (< high school, high school degree, > high school), child weight status, child physical activity, child television viewing, and mother's health status and household smoking. Child physical activity was measured by two questions. The first measured the number of days in the past week that the child exercised or participated in physical activity lasting at least 20 minutes that made him/her sweat and breathe hard. The second question determined whether the child participated in sports lessons or on a sports team in the past year. Television viewing was defined as the number of hours on an average day that a child watches television, videos or plays video games. Mother's health was included as a series of dichotomous dummy variables (poor health, fair health, good health, or very good health with excellent health as the referent). Household smoking was defined as a dichotomous variable indicating whether “anyone in the household uses cigarettes, cigars, or pipe tobacco”.

In general the rationale for including the above covariates was to adjust for previously identified risk factors for asthma and potential confounders. However, given the cross-sectional design of the survey, it is impossible to establish the temporal relationship of these factors. Consequently, we report the unadjusted and fully adjusted models to give a perspective on the potential confounding due to observed covariates, and build models in sequential steps from being most to least parsimonious.

### Analysis

All analyses used the provided sampling weights developed to provide nationally representative estimates [28]. Final multivariable analyses were conducted using STATA to ensure control of the study design effects, proper analysis of the five imputed datasets and correct estimates of standard errors [29], [30], [31]. We first estimated the unadjusted effect of neighborhood safety on the log odds of reporting lifetime asthma and then sequentially adjusted the effect estimate for socio-demographic variables (sex, age, race/ethnicity, SES and child weight status), followed by household variables related to household smoking and mother's health and finally child behavioral variables associated with physical activity and TV viewing.

### Ethics

Verbal consent for participation in the study was obtained from NSCH respondents. Respondents were informed about the voluntary nature of the survey, the authorizing legislation, and confidentiality of data collected. In addition, the informed consent script provided information about the content of the survey and the expected duration. The informed consent was verbal since the survey was via telephone. The study was reviewed by Harvard School of Public Health Institutional Review Board and was considered as exempt from full review as the study was based on an anonymous public use data set with no identifiable information on the survey participants.

## Results

The gender distribution of the study population was relatively equal. A majority of the children were recorded as non-Hispanic white (67.5%), followed by non-Hispanic black (16.1%), White or Black Hispanic (8.0%), and other non-Hispanic (4.6%). The majority of children (65.8%) were in households where at least one resident had more than a high school education. Seventeen percent of children lived in households that were less than 100% of the poverty level. Approximately 15.5% were at risk for overweight (BMI≥85^th^ percentile and <95^th^ percentile) and 21% were overweight (BMI≥95^th^ percentile). Almost half of parents were minimally concerned about neighborhood safety (48.6%), while 16.1% were most concerned (Table 1).

**Table 1:** Sample distribution across exposure and different covariates along with prevalence of reported lifetime asthma, National Survey of Children's Health (Children ages 5–17)*.

Variables	Frequency (%)	Number (%) of total ever diagnosed with asthma
Total population	74,264 (100)	10,271 (14.0)
**Neighborhood Safety**		
Neighborhood sometimes or never safe	8,809 (16.1)	1,492 (18.9)
Neighborhood usually safe	27,257 (35.4)	3,913 (37.1)
Neighborhood always safe	37,299 (48.6)	4,745 (43.9)
**Child demographics**		
**Gender**		
Female	36,039 (48.9)	4,339 (11.8)
Male	38,156 (51.1)	5,925 (16.2)
**Race/ethnicity**		
White Hispanic	4,022 (7.3)	522 (12.1)
Black Hispanic	352 (0.7)	67 (17.1)
Multiple race Hispanic	289 (0.3)	54 (15.3)
Other race Hispanic	358 (0.5)	67 (18.1)
White non-Hispanic	51,828 (67.5)	6,704 (13.3)
Black non-Hispanic	7,176 (16.1)	1,394 (19.1)
Multiple race non-Hispanic	2,608 (3.1)	465 (17.2)
Other race non-Hispanic	2,742 (4.6)	413 (14.3)
**Weight Status of Child**		
Underweight	4,475 (6.8)	498 (12.0)
Normal Weight	40,637 (56.8)	5,250 (13.2)
At-Risk for Overweight	10,672 (15.5)	1,633 (14.9)
Overweight	13,216 (21.0)	2,354 (18.6)
Child on sports team or took sports lessons in past year	42,926 (57.6)	5,879 (14.1)
**Household characteristics**		
**Derived Poverty level** (# range based on imputed datasets)		
Less than 100% of the poverty level	8,335–8,396 (16.8)	1,397–1,415 (15.4)
100% to <200% of the poverty level	14,756–14,797 (22.7)	2,101–2,111 (14.1)
200% to <400% of the poverty level	27,213–27,324 (33.6)	3,592–3,621 (13.7)
≥400% of the poverty level	23,796–23,923 (26.9)	3,146–3,173 (13.4)
**Education**		
Highest Household education is<high school	3,173 (7.6)	386 (11.8)
Highest Household education is = high school	15,584 (26.6)	2,271 (14.0)
Highest Household education is > high school	55,235 (65.8)	7,583 (14.3)
**Smoking**		
Anyone in household use cigarettes, cigars, or pipe tobacco	22,334 (30.3)	3,377 (15.3)
**Mother's health status**		
Mother in poor health	1,340 (2.3)	335 (23.6)
Mother in fair health	5,225 (8.9)	1,021 (18.2)
Mother in good health	15,682 (24.6)	2,488 (15.3)
Mother in very good health	23,566 (32.1)	3,235 (14.1)
Mother in excellent health	22,829 (32.1)	2,408 (10.8)

* unweighted numbers, weighted percents.

There was an inverse association between perceptions of neighborhood safety and the odds of reporting asthma among children (Table 2). In unadjusted models, children living in neighborhoods that were perceived to be sometimes or never safe had substantially higher levels of reported asthma (OR 1.36, 95% CI 1.21, 1.53) as compared to children living in neighborhoods that were perceived to be always safe. The odds ratios for reporting lifetime asthma among children living in neighborhoods that were perceived to be sometimes or never safe decreased to 1.27 (95% CI 1.11, 1.45) after adjustment for demographic and social covariates. Additionally controlling the models for exposure to second hand tobacco smoke in the household as well as mother's self-rated health reduced the odds of reported asthma associated with living in most unsafe neighborhoods to 1.23 (95% CI 1.08, 1.41). Further inclusion of behavioral variables associated with physical activity and television viewing did not attenuate the effect any further (OR 1.25, 95% CI 1.08–1.43).

**Table 2:** Odds ratios (OR) and 95% confidence intervals (CI) for reporting lifetime asthma among children aged 5–17 for exposure and covariates.

Variable	Model 1^a^ (Unadjusted)	Model 2^b^ (Adjusted for sex, age, race/ethnicity, poverty, education and child weight status)	Model 3^c^ (Model 2 additionally adjusted for mother's health status and household smoking)	Model 4^d^ (Model 3 additionally adjusted for children's TV viewing and physical activity)
**Neighborhood**				
Always safe	Reference	Reference	Reference	Reference
Usually safe	1.19 (1.10–1.28)	1.19 (1.10–1.30)	1.14 (1.05–1.24)	1.15 (1.06–1.26)
Sometimes or never safe	1.36 (1.21–1.53)	1.27 (1.11–1.45)	1.23 (1.08–1.41)	1.25 (1.08–1.43)
**Gender**				
Female		0.69 (0.64–0.75)	0.67 (0.62–0.73)	0.68 (0.62–0.74)
Male		Reference	Reference	Reference
**Child age**		1.02 (1.01–1.03)	1.02 (1.01–1.03)	1.02 (1.00–1.03)
**Race/ethnicity**				
White non-Hispanic		Reference	Reference	Reference
White Hispanic		0.99 (0.82–1.20)	1.01 (0.83–1.24)	1.03 (0.84–1.27)
Black Hispanic		1.29 (0.79–2.09)	1.00 (0.58–1.73)	1.10 (0.62–1.94)
Black non-Hispanic		1.40 (1.25–1.57)	1.36 (1.20–1.53)	1.38 (1.22–1.57)
fMulti race Hispanic		1.20 (0.69–2.09)	0.99 (0.54–1.84)	0.88 (0.49–1.57)
Multi race non-Hispanic		1.30 (1.07–1.59)	1.19 (0.98–1.44)	1.18 (0.97–1.45)
Other race Hispanic		1.71 (0.92–3.20)	1.83 (0.94–3.53)	1.81 (0.91–3.62)
Other race non-Hispanic		0.97 (0.75–1.27)	1.07 (0.82–1.41)	1.05 (0.81–1.37)
**Body mass index**				
Underweight		0.95 (0.79–1.13)	0.99 (0.82–1.19)	1.00 (0.82–1.22)
Normal Weight		Reference	Reference	Reference
At risk for overweight		1.15 (1.03–1.28)	1.13 (1.01–1.26)	1.15 (1.03–1.29)
Overweight		1.45 (1.30–1.60)	1.38 (1.24–1.54)	1.38 (1.24–1.55)
**Child participates in sports**				0.97 (0.89–1.07)
**Child physical activity**				1.00 (0.98–1.02)
**TV viewing**				0.98 (0.95–1.00)
**Household poverty**				
<100% of the poverty level		Reference	Reference	Reference
100-<200% of poverty level		0.86 (0.74–1.00)	0.92 (0.79–1.08)	0.93 (0.79–1.09)
200-<400% of poverty level		0.80 (0.70–0.92)	0.91 (0.79–1.05)	0.93 (0.80–1.08)
≥400% of poverty level		0.80 (0.69–0.92)	0.96 (0.83–1.12)	0.97 (0.83–1.14)
**Education**				
Household education less than highschool		0.84 (0.65–1.09)	0.71 (0.55–0.93)	0.72 (0.55–0.95)
Household education equal to high school		0.89 (0.80–0.98)	0.84 (0.76–0.94)	0.85 (0.76–95)
Household education more than high school		Reference	Reference	Reference
**Smoking**				
Presence of Household smoking			1.09 (0.99–1.19)	1.09 (1.00–1.20)
**Maternal self-rated health**				
Mother's health excellent			Reference	Reference
Mother's health very good			1.33 (1.21–1.48)	1.34 (1.21–1.48)
Mother's health good			1.46 (1.30–1.64)	1.45 (1.28–1.63)
Mother's health fair			1.75 (1.48–2.08)	1.70 (1.42–2.02)
Mother's health poor			2.29 (1.78–2.95)	2.28 (1.75–2.95)

a Model 1 was unadjusted.

b Model 2 was adjusted for sex, age, race/ethnicity, poverty, education and child weight status.

c Model 3 was adjusted for sex, age, race/ethnicity, poverty, education and child weight status, mother's health status and household smoking.

d Model 4 was adjusted for sex, age, race/ethnicity, poverty, education and child weight status, mother's health status and household smoking, children's TV viewing and physical activity.

Other significant associations for the odds of reported lifetime asthma in the fully adjusted model were found with gender, race/ethnicity, child's weight, and mother's health (Table 2, Model 4). Female children had a substantially lower likelihood of being diagnosed with asthma. Black non-Hispanic children were substantially more likely to be reported with lifetime asthma compared to white non-Hispanic children. Children who were overweight were also more likely to be reported with lifetime asthma compared to children who were in the ‘normal’ weight range. Poor self-reported health status of mother was strongly associated with reported lifetime asthma. Notably, socioeconomic status was not associated with reported lifetime asthma.

## Discussion

Using the most recent, large and nationally representative survey we report a robust inverse association between parental perception of neighborhood safety and reported lifetime asthma among children. Children living in neighborhoods that parents felt were only sometimes or never safe had a 25% increased risk of being reported with lifetime asthma as compared to those living in neighborhoods where parents felt always safe. The effect size observed for this perceived exposure was comparable to the effects observed for non-Hispanic Blacks or for being overweight; both of which have been strong correlates of asthma. Before we interpret the study findings, the following limitations need to be considered.

First, notwithstanding the general challenges of measuring asthma in population-based studies [32], the measurement of asthma in the NSCH has clear limitations. The NSCH measure of asthma prevalence was based on a single question, as opposed to a hierarchy of asthma/wheeze outcomes based on responses to standardized respiratory questionnaires [33], [34]. No effort was made to clinically test for asthma. However, the prevalence estimates observed in NSCH are not very different from other national estimates [1]. Additionally, there were no data on level of allergens in the home. At the same time, the NSCH, by way of collecting extensive social and demographic data (including perception of neighborhood safety), and being nationally representative, provides a unique, if not the only, opportunity to draw descriptive inference on the patterning of asthma risk by exposure to neighborhood stressors in the U.S. The second limitation relates to neighborhood safety data being based on one question about parental perceptions as opposed to objective systematic observation. Arguably, parents' perception could be as important as actual safety data, since they reflect parental attitudes toward the neighborhood and provide an indication of how parent (and child) might interact with the environment [35]. However, their perceptions could also serve as a proxy for other parental characteristics, such as depression or anxiety, which could independently influence child health. However a convenience sample study examined this idea and found no evidence that parental perception of dangerous neighborhoods was linked to maternal depression [36]. Third, given the cross-sectional and observational study design we cannot rule out both reverse causation (*i.e.*, parents with asthmatic/sick kids moving to neighborhoods that are generally more socially stressful and violent) or confounding due to unobserved confounders (*i.e.*, factors that might be a common prior cause of both child's asthma as well as neighborhood safety condition). Notably, an important confounder is parental health that can influence both the child's risk of asthma and can influence selection into certain type of neighborhoods. While mother's subjective health status is not an ideal measure to control for this potential confounding, it certainly improves the faith in the observed finding that we report between perception of neighborhood safety and childhood asthma. Fourth, the low reported response rate in the NSCH does raise concerns related to the representativeness of the survey and the potential for differential bias. In general, telephone survey response rates have been lower than in past decades. Both the NSCH interview completion rate (68.8%), indicating the completion of interviews among households that were reached, and the overall NSCH response rate were similar to other telephone surveys such as the BRFSS, suggesting that the NSCH achieved a response rate typical for current telephone surveys (see http://www.cdc.gov/BRFSS/technical_infodata/2003QualityReport.htm, accessed May 26, 2009). Additionally, the NSCH post-survey weighting procedures attempted to adjust for non-responses to minimize potential bias. Further, in our analysis, we also found that the prevalence estimates of asthma observed in NSCH were not very different from other national estimates [1]. Finally, there is some evidence to suggest that reducing the non-response rate of a survey does not dramatically change the results [37].

Psychosocial factors that can be linked to asthma mortality, morbidity and medication compliance have been well articulated [38]. Exposure to violence (measured here through parental perception of safety) can be a major psychosocial stressor that may impact the pathogenesis of asthma and/or contribute to asthma morbidity by triggering exacerbations through neuroimmunologic mechanisms [13]. Psychological stress has also been posited to intensify asthma symptoms by increasing the body's inflammatory response to asthma triggers, with the hypothalamic-pituitary-adrenal (HPA) axis, the sympathetic-adrenal-medullary (SAM) axis, and the sympathetic (SNS) and parasympathetic (PNS) arms of the autonomic nervous system serving as the biological pathways [39], between stress and bronchoconstriction in asthma [40]. Sustained exposure to violence, or more importantly the chronic *fear* of violence, may provide the trigger that links psychosocial stress and asthma.

Psychosocial stressors also possibly moderate both humoral and cellular immune function, with such alterations predisposing the individual to respiratory tract infections [41], [42], which may in turn trigger acute asthma episodes. Thus, stress hormones through their influence on immune expression may increase a genetically predisposed individual's risk of developing asthma or perpetuate an existing condition. Violence as a psychosocial stressor, therefore, may be an “adjuvant” to the asthmatic inflammatory response [23]. Current knowledge supports the notion that environmental factors that include viral infection, air pollutants, maternal smoking, breast-feeding, and allergen exposure modulate the expression of the asthmatic phenotype, as related to the immune response. Stress may also accentuate the response to allergens by increasing the release of inflammatory mediators and the subsequent cascade of inflammatory events characteristic of chronic asthma.

Other indirect mechanisms through which exposures to violence (and perhaps other characteristics of stressful social circumstances) may operate is by adopting coping behaviors such as smoking, thus increasing the exposure to a known environmental asthma trigger. For instance, children exposed to second-hand tobacco exposure were at an increased risk for lifetime asthma. However, we were not able to further explore the duration or amount of household smoking because of limitations of the survey data. Nonetheless, we were able to include information from basic questions about household smoking and physical activity, thus controlling for two potential mediators, and a significant effect remained. This suggests that these are not operating as strong mediators in this case. There is some evidence to suggest that even pre-natally, the mother's stress level can influence the programming of the infant's HPA axis and may lead to dysregulation of the infant's immune system [43]. In the case when the stress is caused by fear of violence, such psychosocial exposures may be considered a social determinant of health that actually results in long-term biological changes that contribute to asthma morbidity.

Wolf and colleagues explored biological reasons to explain the association between asthma and parental stress and depression by examining how well these variables could predict children's inflammatory profiles. They found that in both healthy children and children with asthma, parental stress and to a lesser degree, parental depression was associated with changes in the children's inflammatory markers. Specifically, stimulated interlukin 4 (IL-4) production and eosinophil cationic protein (ECP) release significantly increased. These specific markers are relevant to asthma because they are both important parts of the process that leads to an inflammatory event which results in typical symptoms of asthma, such as the constriction of the airway and edema [44].

In addition to this biological response, living in an area plagued by violence can reduce the availability of and access to resources such as doctors, pharmacies and other health care providers. This could make it difficult to successfully manage a child's asthma. It may also lead to negative behavioral changes that are used as coping tools such as parental or child smoking, or an increased indoor and sedentary lifestyle. An increase in sedentary lifestyle could put children at risk for obesity, which is associated with asthma. Indoor living also leads to higher exposure to indoor allergens and exposures that exist in poor quality housing. Additionally, the stress of living in a violent environment may lead to impaired daily functioning, social isolation, feelings of lack of control, and dysfunctional family environment. This may leave families less able to care for asthmatic children [17], [26]. Restriction in socialization could result in increased isolation and decreased social networks and social support which might otherwise serve to ameliorate the effects of community violence. At the same time, because of the cross-sectional design and inability to demonstrate *how* neighborhood perceptions of safety and asthma are related, the findings from this study need replication in other settings. Further, the causal mechanism posited in the preceding discussion also remains to be tested.

The public health relevance of asthma at the national level is well recognized. While physical environmental factors, supplemented with evidence from gene-environment interaction studies, have advanced our mechanistic understanding of this complex disease, they do not fully account for the substantial variation in asthma. To our knowledge, this study is the first to show in a nationally representative sample an inverse association between perceptions of neighborhood safety and childhood lifetime asthma. Stress-induced mechanisms, partially captured through exposure to stressful psychosocial circumstances, may be a critical explanatory link in furthering our understanding of disparities in asthma.
